# Analyzing dietary exposure to critical nutrients on a plant-based diet using the food- and total nutrient index

**DOI:** 10.1186/s12937-025-01105-9

**Published:** 2025-03-12

**Authors:** Maximilian Andreas Storz, Frieda Stübing, Luciana Hannibal, Roman Huber

**Affiliations:** 1 Department of Internal Medicine II, Centre for Complementary Medicine, Medical Center – University of Freiburg, Faculty of Medicine, University of Freiburg, Freiburg, Germany; 2 Faculty of Medicine, Medical Center, Laboratory of Clinical Biochemistry and Metabolism, Department of General Pediatrics, Adolescent Medicine and Neonatology, University of Freiburg, Freiburg, Germany

**Keywords:** Total Nutrient Index, Micronutrients, Potassium, Plant-Based Diet, Choline, Vitamin A

## Abstract

**Background:**

Unfortified plant-based diets are devoid of vitamin B12, and supply low intakes of iodine, zinc, selenium, and calcium. To disentangle the complex interplay between nutritional adequacy and nutrient intakes from supplements and foods in plant-based diets, data from a Germany-based cross-sectional study examining the nutritional status of omnivores, lacto-ovo-vegetarians and vegans was re-analyzed. Special emphasis was put on potentially under-consumed nutrients in plant-based diets, including vitamin A and choline.

**Methods:**

A novel tool focusing on under-consumed micronutrients was employed to shed a new light on nutrient supply and dietary exposure to critical nutrients in plant-based diets: The Total Nutrient Index (TNI). The TNI extends existing measures of diet quality by considering nutrient intake data from both foods *and* supplements. The TNI covers calcium, magnesium, potassium, choline and vitamins A, C, D, and E. The TNI was compared between omnivores, vegetarians and vegans, with a special focus on its micronutrient component scores and with regard to dietary supplement contributions.

**Results:**

Data from 108 participants was analyzed. The vegan and the omnivorous diet resulted in similar TNI scores (73.70 ± 19.68 and 72.77 ± 17.88), whereas lacto-ovo-vegetarians scored lower (68.50 ± 17.10). The contribution of supplements to the TNI was higher in vegans and omnivores (median contribution: 12.50 (16.80) and 10.81 (18.23) score points, respectively) as compared to lacto-ovo-vegetarians (3.42 (12.50) score points). High micronutrient component score contributions to the TNI were found for vitamin D supplements (all dietary groups), vitamin C supplements (omnivores and vegans) and magnesium supplements (all groups).

**Conclusions:**

Supplementation has a profound impact on nutrient supplies in individuals on a plant-based diet. This study reiterates the need to quantitatively assess nutrient intakes from supplements to assess diet quality of plant-based dietary patterns. We posit that defining diet-specific TNI scores is important for a precise evaluation of diet quality, whether in omnivore or in the spectrum of plant-based diets.

**Supplementary Information:**

The online version contains supplementary material available at 10.1186/s12937-025-01105-9.

## Background

The lacto-ovo-vegetarian diet and the vegan diet are currently among the most popular plant-based dietary patterns in Germany and many other European countries [1–3]. Lacto-ovo-vegetarians consume milk and dairy products but avoid flesh foods. Vegans, on the other hand, exclude all animal products from their menus [1, 2].

Plant-based dietary patterns have been associated with a more favorable lipid intake and higher intakes of fiber, complex carbohydrates, phytochemicals, magnesium and potassium [4–7]. Of note, unhealthy and unbalanced vegetarian/vegan diets, which are centered around foods rich in refined carbohydrates, high-fructose corn syrup, saturated fatty acids and artificial sweeteners, may also predispose an individual to micronutrient deficiencies [4]. Potentially critical nutrients and nutrient inadequacies in plant-based diets are subject to an ongoing scientific debate [8–10].

This debate is often centered around vitamin B12, vitamin D, calcium, iodine and zinc [11, 12]. Based on several recent publications, however, other important and potentially *under-consumed* nutrients (e.g., specific amino acids and omega-3-fatty acids) are seldom part of that discussion, and possibly received too little attention in the past [13, 14].

This may apply in particular to choline and vitamin A, which—depending on an individual’s diet quality—are potentially lacking in a plant-based diet [8, 9, 15, 16]. Major dietary choline sources include eggs and low-fat milk, although choline may also be obtained in larger amounts from green leafy vegetables and potatoes [17]. Vitamin A may be obtained from various sources, either in the form of preformed vitamin A (obtained through dairy products and meat) or in the form of provitamin A carotenoids (obtained through legumes, nuts, seeds, grain products, fruits and vegetables) [18].

A previous cross-sectional study conducted by our team compared the nutritional status of young, healthy vegans, lacto-ovo-vegetarians and omnivores in Southern Germany [8, 9]. While that particular work was centered around vitamin B12 status, concerns were also raised with regard to vitamin A [8, 9]. These concerns were reinforced by a recent position paper of the German Nutrition Society regarding vegan diets, which now also lists vitamin A as a potentially critical nutrient [15].

This project was revisited in the form of a secondary analysis and choline intakes were additionally computed which was not done previously. Dietary vitamin A intake in retinol activity equivalents was also recalculated based on an additional nutrient intake database.

A novel nutrient-based dietary index focusing on eight potentially *under-consumed* micronutrients was employed to gain additional insights into nutrient intakes in plant-based diets: the Total Nutrient Index (TNI) by Cowan et al. [19, 20]. The TNI extends existing measures of diet quality by considering nutrient intake data from all sources, including foods *and* supplements [19, 20]. The major aim was to shed a new light on dietary exposure to critical nutrients in a plant-based diet in this cohort, and to disentangle the complex interplay between nutritional adequacy, and nutrient intakes from supplements, and foods, respectively.

Based on previous insights into nutrient intakes in this cohort [8, 9], it was hypothesized that substantial differences in TNI scores would exist between vegans, lacto-ovo-vegetarians and omnivores, with both plant-based dietary patterns faring worse than the omnivorous diet, potentially due to lower intakes of vitamin A and choline. A secondary aim of the study was to determine the extent to which supplements contributed to nutrient intakes and the TNI, for which previously reported supplement intake frequencies and dosages were re-analyzed to estimate diet group-specific mean/median intakes per year.

## Methods

### Study population and design

The study population has been described elsewhere in great detail [8, 9]. In brief, the main objective of this study was to compare nutrient intake data and supplementation behavior in adult and healthy omnivores, lacto-ovo-vegetarians and vegans (diet adherence: > 2 years) based in Southern Germany. Nutrition biomarkers regarding participants’ vitamin B12 status, physical activity data and other sociodemographic variables were captured in a cross-sectional design and compared between the 3 groups. Four-day weighed food diaries as well as in-person supplement intake assessments were used to assess nutrient intake data from both foods and supplements [8, 9]. A special emphasis in this study was laid to supplementation intake and behavior in participants to explore the contribution of nutrients from dietary supplements to the overall nutrient intake.

### The total nutrient index and food nutrient index

The TNI and Food Nutrient Index (FNI) were originally created by Cowan et al. to assess the total micronutrient exposure in US adults [19, 20]. The TNI is unique in a way that it—unlike other dietary indices—includes exposures from dietary supplements, which may provide substantial amounts of micronutrients [19, 20]. The TNI is particularly valuable when assessing total nutrient exposures of eight *under-consumed* micronutrients (see below), and has been validated previously using data from the US-based National Health and Nutrition Examination Surveys [19].

When assessing under-consumed micronutrients in plant-based diets, it was deemed necessary to account for the high prevalence of dietary supplement usage among plant-based individuals [21, 22], and their large contribution to the total nutrient intake. In the herein presented cohort, approximately 92% of vegans and 51% of lacto-ovo-vegetarians used dietary supplements [8, 9]. The TNI extends existing measures of diet quality by considering nutrient intake data from all sources [19, 20]. This is of particular importance in the present cohort, in which supplement usage was a widespread phenomenon [8, 9]. This approach also increases precision, as dietary supplements in studies on plant-based diets were often captured on a qualitative basis only [8, 21, 22].

The TNI assesses nutrient intakes relative to the recommended dietary intakes and adequate intakes in the Dietary Guidelines for Americans (DGA) [10, 19, 23, 24]. Age and sex-specific nutrient intake recommendations used for the TNI assessment in this study are listed in Supplementary Table 1. As discussed earlier, the TNI focuses on selective *under-consumed* micronutrients, namely vitamins A, C, D, and E, calcium, magnesium, potassium, and choline. The scoring algorithm has been described elsewhere in great detail [10, 19]. In brief, the TNI is scored from 0 to 100 and truncated at 100% of the respective standard [19, 23]. The higher the TNI, the closer the alignment with the nutrient intake recommendations found in the DGA [19, 23, 24]. The overall score is the average of the eight equally weighted micronutrient component scores.

The TNI has two sub-components: the FNI (which is calculated identically to the TNI but considers foods only) *and* a second part which considers nutrients from dietary supplements. In light of the purpose of this paper, the FNI and TNI values are presented separately for each dietary group. The score-difference (e.g., the TNI *minus* the FNI) reflects the contribution of dietary supplements in each group. While the TNI was constructed and designed for the United States, it was deemed useful for this Germany-based study in order to gain new insights into critical nutrients on a plant-based diet. The high alignment between the DGA-based nutrient intake recommendations and the national nutrient intake recommendations in Germany (e.g., for calcium and vitamin A) supported its usage in this cohort [25].

### Nutrient intake data from foods

The assessment of nutrient intake data and the involved steps upon the evaluation of the nutritional protocols using NutriGuide® plus software (Version 4.9, Nutri-Science GmbH, Hausach, Germany) has been described earlier in detail [8, 9]. 4-day weighed food diaries were used to estimate nutrient intakes, following an approach described elsewhere [8, 9]. Choline intake values could not be obtained using NutriGuide® plus software and were therefore calculated manually. For this, the USDA (US Department of Agriculture) database for the choline content of common foods and the USDA national nutrient database for standard reference legacy (2018) were used [26, 27]. Several foods that are typically consumed on a plant-based diet could not be retrieved from these databases (e.g. plant-based meat alternatives or dairy alternatives except soy milk) [26, 27]. In such cases, other relevant literature was consulted (e.g., [28]) or, when unavailable, the choline content of said foods was estimated by looking at the individual ingredients and their choline content.

### Nutrient intake data from supplements

Supplement assessment has been discussed earlier in detail [8, 9]. All supplements taken by participants within the last year were registered with their intake frequency and their daily dosage. Based on this data, median intake frequencies in mg or IU were calculated for the whole sample and for supplementing individuals only.

### Inclusion and exclusion criteria

The full original cohort comprised *n* = 115 individuals [8, 9]. For this secondary data analysis, *n* = 7 individuals were excluded. This was done because the FNI and TNI are typically calculated in an age- and sex-specific manner, based on nutrient intake data recommendations in the DGA [19, 20, 24]. Since the herein presented cohort included only *n* = 7 individuals aged 51 years or older, subpopulation statistics for this age group would not have been reliable/feasible. Apart from this aspect, no other exclusion criteria applied and all other participants were considered for this secondary analysis.

### Research ethics

The project was approved by the ethical committee of the University Medical Center of Freiburg, Germany (EK Freiburg 21–1442). The study is registered in the German national trial register under the following code: DRKS00027425.

### Statistical analysis

The statistical analysis was performed in STATA 14 (StataCorp. 2015. Stata Statistical Software: Release 14. College Station, TX: StataCorp LP). Using subpopulation summary statistics and Stata’s Shapiro–Wilk W test for normality, the distribution of the data was examined. Normally distributed variables were presented with their mean ± standard deviation. For non-normally distributed variables, the median and the interquartile range were given. Strip plots and deviation plots were used to visualize the distribution of data points in each group. The user-written Stata command “stripplot” was used to plot data as a series of marks against a single vertical magnitude axis, while also displaying boxes showing group-specific medians and quartiles [29].

For the between group comparisons, parametric and non-parametric tests including the Kruskal–Wallis H test and one-way analysis of variance (ANOVA) were used. When statistically significant differences were identified, a post hoc Dunn’s test was applied to identify between group differences. The chi-square test of association was used to compare differences in categorical variables. Spearman’s rank-order correlations and Pearson's product moment correlation coefficients were used to assess the relationship between the TNI and the serum concentrations of various TNI-relevant vitamins. Box plots, deviation plots [30], scatter plots and separated scatter plots were created to visualize the results. Statistical significance was determined at α = 0.05.

## Results

**Fig. 1:**
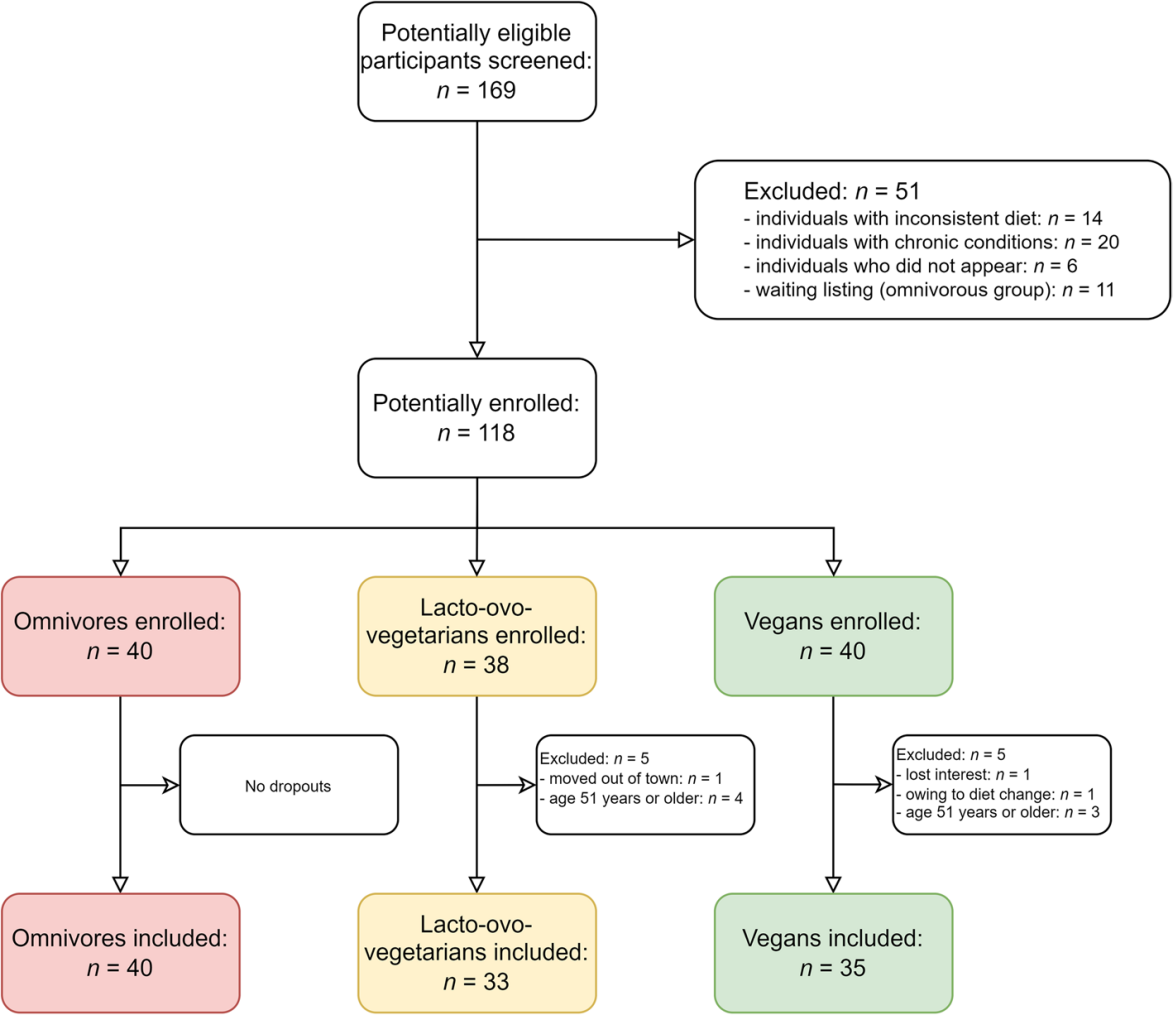
Participant inclusion flowchart. Legend: The final sample comprised *n* = 108 participants. A total of *n* = 7 participants were excluded from this sub-analysis for being older than 50 years (*n* = 4 in the lacto-ovo-vegetarian group and *n* = 3 in the vegan group)

The total subsample for this analysis comprised *n* = 108 participants (Fig. 1, based on [8, 9]). Of these, *n* = 70 participants (64.81%) belonged to the age category 18–30 years; the remaining *n* = 38 (35.19%) individuals belonged to the age category 31–50 years. Figure 1 depicts a participant inclusion flow chart, showing reasons for in- and exclusion of participants. The analyzed sample included *n* = 40 omnivores, *n* = 33 lacto-ovo-vegetarians and *n* = 35 vegans.

Table 1 displays the participants’ sociodemographic and anthropometric data by dietary group. In line with our previous study based on the full cohort [8, 9], no between group differences were found except for the duration of the dietary adherence (336 months in omnivores, 60 months in lacto-ovo-vegetarians and 52 months in vegans (*p* < 0.001)).

**Table 1:** Sample characteristics

	**Omnivores (*n* = 40)**	**Lacto-Ovo-Vegetarians (*n* = 33)**	**Vegans (*n* = 35)**	***p*-value**
**Sex**				0.711 ^a^
Male	*n* = 16 (40%)	*n* = 11 (33.33%)	*n* = 15 (42.86%)	
Female	*n* = 24 (60%)	*n* = 22 (66.66%)	*n* = 20 (57.14%)	
**Age** (years)	30.78 ± 6.97	27 (6)	26 (8)	0.183 ^b^
**Marital Status**				0.537 ^a^
Single	*n* = 29 (72.50%)	*n* = 29 (87.88%)	*n* = 28 (80.00%)	
Married	*n* = 9 (22.50%)	*n* = 4 (12.12%)	*n* = 7 (20.00%)	
Divorced	*n* = 1 (2.50%)	*n* = 0 (0%)	*n* = 0 (0%)	
Other	*n* = 1 (2.50%)	*n* = 0 (0%)	*n* = 0 (0%)	
**Race/ethnicity**				0.350 ^a^
Caucasian	*n* = 40 (100%)	*n* = 33 (100%)	*n* = 34 (97.14%)	
Turk	*n* = 0 (0%)	*n* = 0 (0%)	*n* = 1 (2.86%)	
**Height** (cm)	173.10 ± 9.32	173.42 ± 7.40	174.23 ± 10.07	0.860 ^c^
**Weight** (kg)	70.16 ± 14.63	64 (10)	70 (20)	0.287 ^b^
**Body Mass Index** (kg/m^2^)	22.91 (4.99)	21.49 ± 2.01	22.40 (4.13)	0.068 ^b^
**Dietary adherence** (months)	336 (162)	60 (66)	52.34 ± 25.06	** < 0.001 **^**b**^
**Educational level**				0.973 ^a^
Secondary school	*n* = 4 (10.00%)	*n* = 2 (6.06%)	*n* = 3 (8.57%)	
German Abitur	*n* = 19 (47.50%)	*n* = 15 (45.45%)	*n* = 16 (45.71%)	
University degree	*n* = 17 (42.50%)	*n* = 16 (48.48%)	*n* = 16 (45.71%)	
**MacArthur Scale of Subjective Social Status**	6.38 ± 1.35	6.61 ± 1.20	6.18 ± 1.45	0.860 ^c^

Normally distributed data is shown with its mean ± standard deviation; not normally distributed data is shown with its median and IQR in parenthesis

^a^ = based on Stata’s Chi-Square Test of independence

^b^ = based on Kruskal–Wallis H test

^c^ = based on analysis of variance

No differences between the three examined dietary groups were found for total energy intake and macronutrients intakes in this subsample. Relevant nutrient intake differences from foods between the 3 groups were found for fiber and choline (*p* = 0.002 for both) as well as vitamin C and vitamin D (*p* < 0.001 for both) (Table 2). For all 4 nutrients, intakes between omnivores and vegetarians and between omnivores and vegans differed significantly (*p* < 0.01 for all, as per Dunn’s test). Dunn’s test suggested no significant intake differences between vegans and vegetarians.

**Table 2:** Energy, macro- and micronutrient intake from foods

	**Omnivores (*n* = 40)**	**Lacto-Ovo-Vegetarians (*n* = 33)**	**Vegans (*n* = 35)**	***p*-value**
Energy intake (kcal/day)	2229.61 ± 706.79	2085.86 (662.43)	2071.14 (887.29)	0.868 ^a^
**Macronutrients**				
Carbohydrate intake (g/day)	244.60 ± 75.34	241.75 (102.15)	250.28 (140.86)	0.223 ^a^
Fat intake (g/day)	81.39 (49.27)	75.85 (35.77)	88.74 ± 31.82	0.994 ^a^
Protein intake (g/day)	73.66 (45.43)	65.88 (24.71)	61.85 (42.82)	0.312 ^a^
**Micronutrients**				
Calcium intake (mg/day)	541.24 (448.97)	548.64 (230.95)	483.93 (192.03)	0.322 ^a^
Choline intake (mg/day)	328.94 (191.47)	236.82 (88.35)	257.35 (100.02)	**0.002** ^a^
Fiber intake (g/day)	26.11 ± 12.73	29.59 (12.77)	33.45 (19.54)	**0.002** ^a^
Magnesium intake (mg/day)	246.76 (177.96)	291.57 (107.21)	313.49 (170.82)	0.065 ^a^
Potassium intake (mg/day)	2155.03 ± 951.16	2221.88 ± 687.38	2559.63 (1198.09)	0.103 ^a^
RAE intake (μg/day)	466.63 (474.60)	424.40 (199.64)	338.37 (319.54)	0.337 ^a^
Vitamin C intake (mg/day)	103.21 (67.19)	156.70 ± 83.70	186.09 ± 96.85	** < 0.001** ^a^
Vitamin D intake (IE/day)	82.80 (120)	49.14 (58.63)	35.43 (39.09)	** < 0.001** ^a^
Vitamin E intake (mg/day)	9.74 (9.80)	10.44 (11.64)	13.68 (13.01)	0.234 ^a^

RAE = retinol activity equivalents. Normally distributed data is shown with its mean ± standard deviation; not normally distributed data is shown with its median and IQR in parenthesis

^a^ = based on Kruskal–Wallis H test. Nutrient intakes shown in this table are from foodstuffs only and do not include nutrients taken in the form of supplements. Estimated nutrient intakes from supplements are provided in Table 3

Tables 3 and 4 display nutrient intakes from supplements in the entire sample and in supplementing individuals only.

**Table 3:** Nutrient intake from supplements: whole sample (*n* = 108 participants)

	**Omnivores (*n* = 40)**	**Lacto-Ovo-Vegetarians (*n* = 33)**	**Vegans (*n* = 35)**	***p*-value**
Calcium intake (mg/day)	0 (0)	0 (0)	0 (17.53)	**0.020 **^**a**^
Choline intake (md/d)	0 (0)	0 (0)	0 (0)	0.237 ^a^
Magnesium intake (mg/day)	0 (44.10)	0 (0)	0 (11.40)	0.232 ^a^
Potassium intake (mg/day)	0 (0)	0 (0)	0 (0)	0.427 ^a^
Vitamin A intake (µg/day)	0 (0)	0 (0)	0 (0)	0.223 ^a^
Vitamin C intake (mg/day)	0 (37.26)	0 (0)	0 (0)	0.068 ^a^
Vitamin D intake (IU/day)	142.47 (998.63)	57.53 (986.30)	493.15 ( 1780.82)	0.343 ^a^
Vitamin E intake (mg/day)	0 (0)	0 (0)	0 (0)	0.494 ^a^

Nutrient intake data from supplements was not normally distributed and is shown with its median and IQR in parenthesis

^a^ = based on Kruskal–Wallis H test

**Table 4:** Nutrient intake from supplements: supplementing participants only

	**Number of supplementing individuals**	**Omnivores**	**Lacto-Ovo-Vegetarians**	**Vegans**	***p*-value**
Calcium intake (mg/day)	*n* = 14	*n* = 3; 131.51 (75.37)	*n* = 2; 53.84 (92.33)	*n* = 9; 100 (100)	0.373 ^a^
Choline intake (mg/d)	*n* = 12	*n* = 2; 82.88 (34.25)	*n* = 4; 29.31 (57.67)	*n* = 6; 100 (50)	0.279 ^a^
Magnesium intake (mg/day)	*n* = 33	*n* = 16; 69.74 (158.01)	*n* = 7; 49.86 (303.65)	*n* = 10; 75 (151.75)	0.965 ^a^
Potassium intake (mg/day)	*n* = 1	*n* = 1; 705 (0)	*n* = 0	*n* = 0	
Vitamin A intake (µg/day)	*n* = 9	*n* = 3; 329.42 (186.30)	*n* = 1; 668 (0)	*n* = 5; 450 (300)	0.110 ^a^
Vitamin C intake (mg/day)	*n* = 25	*n* = 14; 58.30 (125.48)	*n* = 4; 200 (585)	*n* = 7; 32.05 (62.90)	0.186 ^a^
Vitamin D intake (IU/day)	*n* = 65	*n* = 25; 797.81 (1178.08)	*n* = 18; 806.58 (980.61)	*n* = 22; 1181.94 (1932.33)	0.149 ^a^
Vitamin E intake (mg/day)	*n* = 7	*n* = 4; 6.95 (7.65)	*n* = 1; 30 (0)	*n* = 2; 4.92 (3.84)	0.300 ^a^

Nutrient intake data from supplements was not normally distributed data is shown with its median and IQR in parenthesis

^a^ = based on Kruskal–Wallis H test. Table 4 uses the following format: n (number of observation in each dietary group), median (IQR)

**Fig. 2:**
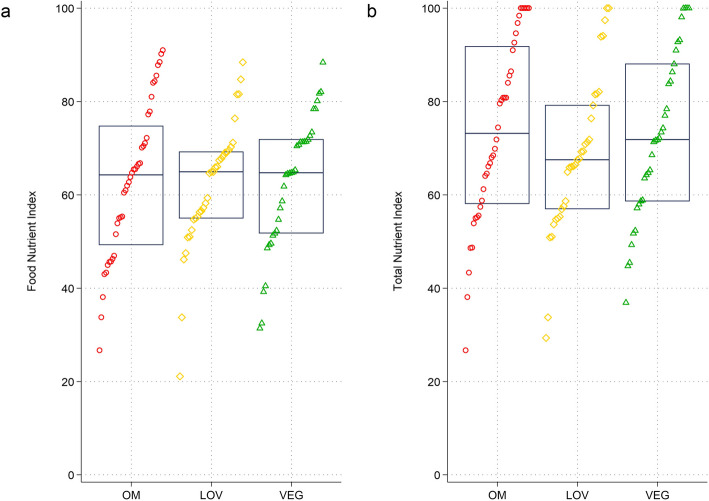
Strip plots – Food Nutrient Index (FNI) and Total Nutrient Index (TNI) by dietary group. Legend: Strip plots showing the FNI (panel **a**), and TNI (panel **b**) by dietary group. Both scores range from 0 to 100. Based on *n* = 108 observations. OM = omnivores; LOV = lacto-ovo-vegetarians; VN = vegans. For a better overview, individual observations are displayed in red circles (omnivores), yellow orange rhombuses (vegetarians) or green triangles (vegans)

The FNI and TNI range and dispersion of observations within each diet category is shown in Fig. 2. No between group differences were found (*p* = 0.954 and 0.792, respectively). As for the TNI, omnivores had the highest score (73.70 ± 19.68), followed by vegans (72.77 ± 17.88) and lacto-ovo-vegetarians (68.50 ± 17.10). Supplementary Table 2 displays FNI and TNI scores by dietary group and by age group. Supplementary Fig. 1 displays ANOVA-based deviation plots and unadjusted deviation plots, allowing for additional insights into the group-specific FNI and TNI distribution and visualizing deviations from the mean in an increasing order.

**Fig. 3:**
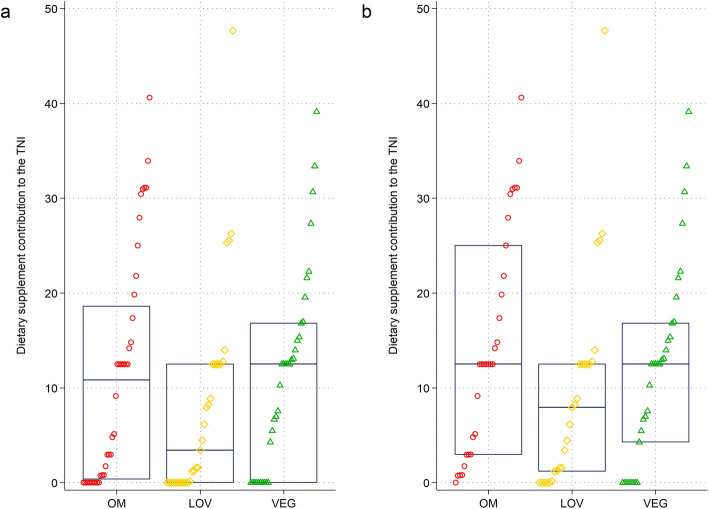
Strip plots – contribution of dietary supplements to the Total Nutrient Index (TNI) by dietary group. Legend: Strip plot showing the contribution of dietary supplements to the TNI in the entire sample (panel **a**) and in supplementing individuals only (panel **b**). The herein visualized score-difference (e.g., the TNI *minus* the FNI) reflects the contribution of dietary supplements in each group. The contribution of supplements may range from 0 to 100 points. Based on *n* = 108 observations. OM = omnivores; LOV = lacto-ovo-vegetarians; VN = vegans. For a better overview, individual observations are displayed in red circles (omnivores), yellow orange rhombuses (vegetarians) or green triangles (vegans)

**Fig. 4:**
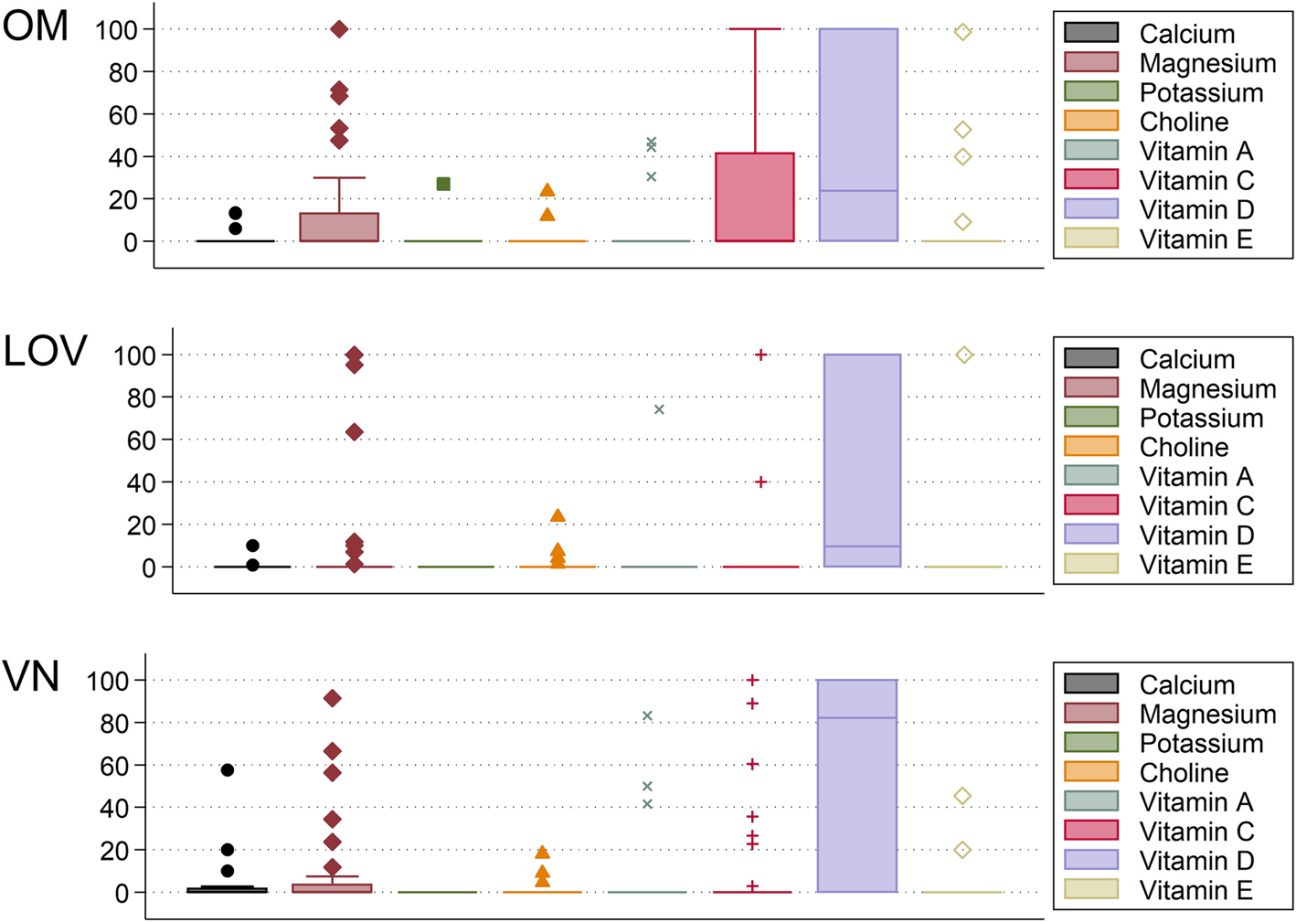
Dietary supplement contribution to the TNI by dietary group and nutrient. Legend: Fig. 4 shows box plots depicting the contribution of dietary supplements to TNI-relevant nutrients in the 3 examined dietary groups (top = omnivores (OM); middle = lacto-ovo-vegetarians (LOV); bottom = vegans (VN)). Box limits indicate the range of the central 50% of the data, with a central line marking the median. Lines extending from each box capture the range of the remaining data, with separate dots indicating outliers. Based on *n* = 108 observations

Figure 3 displays the contribution of dietary supplements to the TNI score by dietary group. The overall contribution of supplements to the TNI was almost equal in omnivores (median contribution: 10.81 (18.23) score points in all participants) and vegans (median contribution: 12.50 (16.80) score points in all participants), whereas it was less pronounced in lacto-ovo-vegetarians (median contribution: 3.42 (12.50) score points in all participants). When looking at individual micronutrients (Fig. 4), important median TNI score point contributions by supplements were found for magnesium (20.93 (43.86) in supplementing omnivores, 11.87 (88.10) in supplementing vegetarians and 17.86 (50.64) in supplementing vegans), vitamin C (71.89 ( 61.64) in supplementing omnivores and 35.62 (66.09 in supplementing vegans), and vitamin D (100 (78.08) in supplementing omnivores, 50 (100) in supplementing vegetarians and 54.79 (100) in supplementing vegans).

**Fig. 5:**
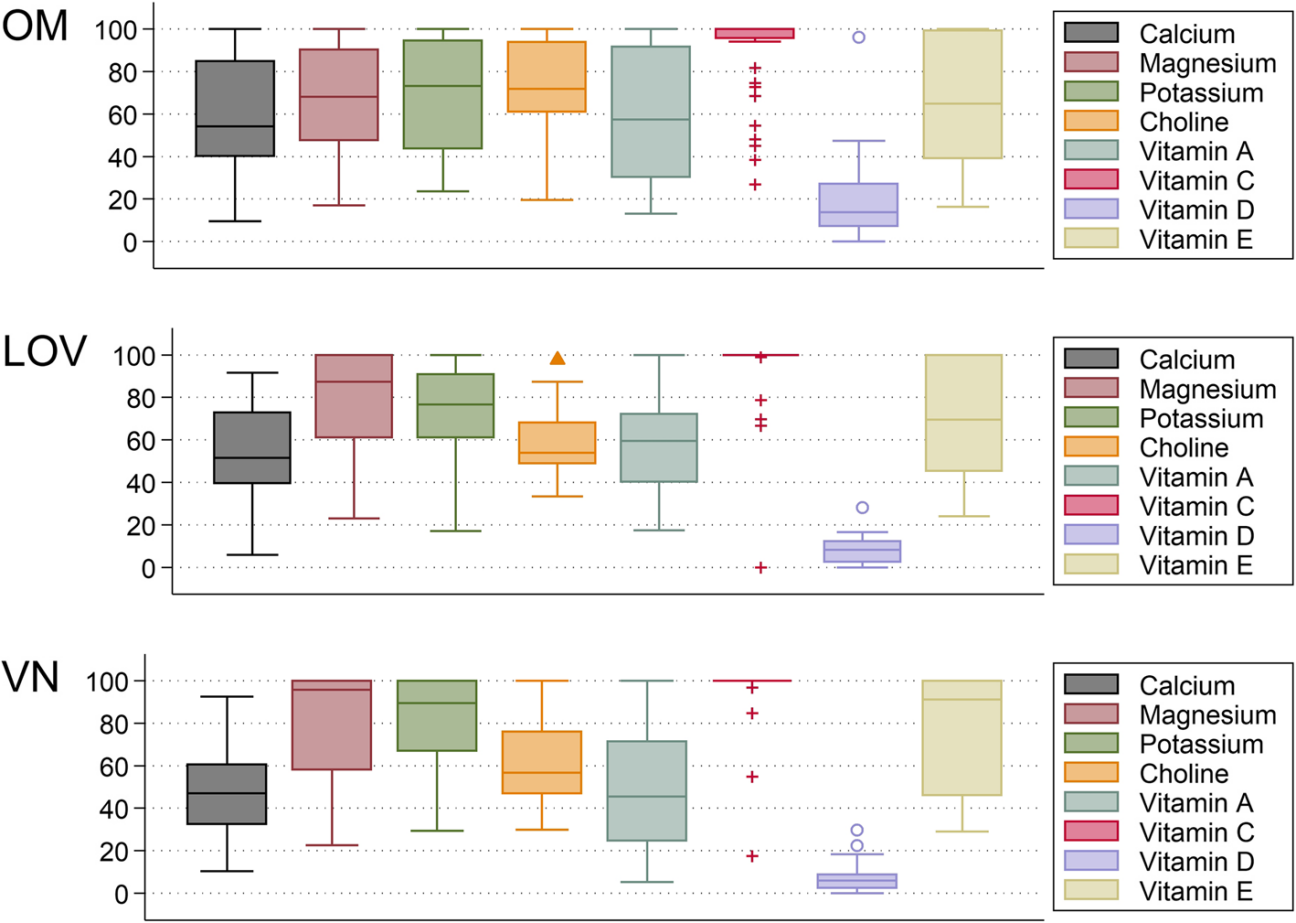
Variation in Food Nutrient Index (FNI) components by dietary group. Legend: Fig. 5 shows a series of box plot of FNI-relevant nutrients in the 3 examined dietary groups (top = omnivores (OM); middle = lacto-ovo-vegetarians (LOV); bottom = vegans (VN)). Based on *n* = 108 observations. Box limits indicate the range of the central 50% of the data, with a central line marking the median. Lines extending from each box capture the range of the remaining data, with separate dots indicating outliers

The group-specific variations in singular FNI components by dietary group are shown in Fig. 5. Relevant between group differences were found for the following components: magnesium (*p* = 0.037), choline (*p* = 0.002), and vitamin D (*p* =  < 0.001). As for magnesium, the contribution in vegans differed significantly from omnivores (*p* = 0.006) but not from vegetarians (*p* = 0.05). For choline and vitamin D, contributions differed between omnivores and vegetarians (*p* =  < 0.001 for both) and between omnivores and vegans (*p* = 0.004 and < 0.001, respectively). As per Dunn’s test, no significant differences were found between vegetarians and vegans for all 3 nutrients (*p* = 0.20, 0.19 and 0.22, respectively).

Finally, Spearman’s rank-order correlations were ran to identify potential associations between the TNI and the serum levels of various vitamins. A significant correlation was found for vitamin D (Spearman's rho = 0.45; *p* =  < 0.001, based on) and vitamin A (Spearman's rho = 0.21; *p* = 0.034), whereas the other associations were not significant (Supplementary Fig. 2).

## Discussion

The indexing of what constitutes critical nutrients on a plant-based diet is subject to an ongoing scientific debate [31–33]. Plant-based diets have become increasingly popular in many European countries [2]. Recent nationally-representative data suggest that more than eight percent of the German population now follows a lacto-ovo-vegetarian diet, whereas approximately two percent follows a vegan diet [34]. As such, plant-based diets have emerged as a public health nutrition topic requiring thorough attention.

Health and environmental benefits of plant-based diets are being explored actively worldwide [2], and their benefits in terms of the prevention of many non-communicable diseases are widely accepted [2, 8, 10, 12, 33]. Identifying critical nutrients on a plant-based diets is of fundamental importance, firstly to more precisely outline what constitutes *healthy* vs *unhealthy* plant-based diets [35], and secondly to attend to the emergent and potentially unhealthy consumption of highly-processed “plant-based foods” (mainly meat and cheese alternatives), which often do not contribute to an improved nutrient intake [36].

Here we employed a validated nutrient-based dietary score to assess the consumption and supplementation of eight *potentially under-consumed* nutrients on a plant-based diet. Both the vegan and the omnivorous diets resulted in higher TNI scores when compared to the lacto-ovo-vegetarian diet. The contribution of supplements to the TNI was approximately twice as high in vegans and omnivores in comparison to the examined lacto-ovo-vegetarians. When looking at nutrient intakes from foods only (FNI-score), lacto-ovo-vegetarians, omnivores and vegans ranked almost equally.

These subtle but important differences reiterate the need to consider nutrient intakes from supplements when discussing about the diet quality of plant-based dietary patterns. A qualitative supplement intake and behavior assessment (*yes/no*) as often seen in plant-based nutrition studies is insufficient. To the contrary, frequency and dosages of supplements must be meticulously assessed to adequately capture their contribution to the overall diet quality.

All three examined dietary groups were characterized by an insufficient dietary intake of calcium. Omnivores and lacto-ovo-vegetarians had a substantially lower potassium intake in comparison to vegans. The present study reiterates that both nutrients are “nutrients of public health concern”, which require especial attention regardless of the dietary pattern [24]. Notably, the TNI does not reflect the overall dietary quality but focuses on eight selective (potentially *under-consumed*) nutrients. Some nutrients of public health concern are not included in the TNI [20]. This may apply in particular to fiber, which is frequently *under-consumed* by the general population but abundant in plant-based diets focusing on whole-grains, legumes, vegetables and fruits [8, 9, 33]. In this study, TNI scores were comparable across the three dietary groups, yet, they resulted from largely different nutrient intake patterns. This suggests that TNI scores may require definition by dietary type, and as such, they hold limited value as a tool to comparatively assess the quality of different diets.

Contrasting the herein presented results from a cohort of healthy individuals with isocaloric intakes to the data from Cowan et al. based on the US general population [19], FNI scores were lower in non-supplementing omnivores. Lacto-ovo-vegetarians and vegans in this study also ranked below the mean FNI score of non-supplementing omnivores in the Cowan study. A comparable picture was found for the TNI. Here, a reservation must be made, that the TNI is a non-energy-adjusted tool, and a direct comparison between populations and countries does not appear to be feasible for this reason. Food fortification in the United States may also play a role in this context [37].

The micronutrient choline, recently added to the list of “critical nutrients” to calculate the TNI [19], has received renewed attention in particular in women of child-bearing age and pregnant [38]. While choline biosynthesis occurs in the liver [39], the major supply comes from the diet, and it is particularly enriched in foods of animal origin. It follows that choline intake will be expectedly lower in individuals adhering to diets low in animal products. In line with this, higher intake of choline was observed in omnivores when compared to the other two diet groups in this study. This is of particular importance in light of a 2019 editorial by Derbyshire, who emphasized the “*mounting evidence of choline’s importance*”, and highlighted that the “*accelerated food trends towards plant-based diets/veganism could have further ramifications on choline intake/status*” [40]. While intake differences in choline were observed (omnivores had the highest intakes, followed by vegans and lacto-ovo-vegetarians), it must be emphasized that all examined dietary patterns fared almost equally in terms of the overall FNI score. The required daily intake amount of choline is still subject to an ongoing debate and how much dietary choline is precisely necessary is hardly assessable with the currently available data [39]. Moreover, there are negative aspects of surplus choline intake. For instance, excessive choline intake has been associated with prothrombotic effects [41]. Tang et al. emphasized that the production of the proatherosclerotic metabolite trimethylamine-N-oxide from dietary phosphatidylcholine may negatively affect cardiovascular health [42].

As shown in this study, plant-based diets may provide a certain amount of choline; which, on a mean basis, may account for up to 57.23% of the recommended daily intake in lacto-ovo-vegetarians and to 61.27% of the recommended daily intake in vegans. Choline supplementation in in this study was however rare, and found only in 12 (11.11%) participants. In light of the above and the available literature, there is no evidence to support that adhering to a healthful plant-based diet may lead to choline deficiency. It must thus be kept in mind that the inclusion of choline as a critical nutrient introduces a bias in the TNI scores that may equivocally prone to recommendations to increase choline intake, while its necessity is far from being proven in individuals adhering to plant-based diets.

The presented study is not without limitations. We focused on a cohort of healthy young adults, having adhered to omnivore, ovo-lactovegetarian or vegan diets for at least 2 years. The diets had a comparable caloric intake. This does not represent the average demographic or nutritional intakes in Germany. Despite this major limitation and the limited sample size, our secondary data analysis has many strengths, including the detailed dietary assessment (based on weighed food diaries), the detailed supplement assessment (going beyond a qualitative assessment), as well as the ability to differentiate nutrient intakes from both sources. Choline intakes were computed on a manual basis using a large food composition database [26, 27], as it is not routinely included in standard dietary software [40]. While this is per se a strength of the study, it must be clearly emphasized that not all foods reported by our study participants were found in the USDA list. Occasionally, we searched for other relevant literature (e.g., [20]) or, when unavailable, estimated the choline content of said foods by looking at individual ingredients and their choline content. Finally, the TNI is a US-centered nutrient index and was, to the best of our knowledge, so far not used in conjunction with Germany-based cohorts. Nevertheless, the index was deemed useful for its coverage of potentially critical nutrients on a plant-based diet, including calcium, vitamin D and vitamin A. The latter was recently added to the list of potentially critical nutrients on a plant-based diet by the Germany nutrition Society [15]. The TNI is an epidemiological tool that per se does neither consider nutrient interactions nor bioavailability or matrix effects, which would have further added to the quality of this article.

From a statistical and practical point of view, the dietary supplement assessment technically covered a time period (1 year) different from the weighed food records (4-day weighed food diaries). This limitation, alongside the lack of measurement error correction methods found in larger epidemiological studies [43–46], warrants careful consideration when interpreting the results. At this stage, current methods to estimate usual intakes are not designed specifically to handle dietary supplements [44]. A “shrink then add” approach as described by Bailey would have added to the methodological strength of this analysis [44]. Likewise, TNI scores were computed from data that may not reflect usual intakes, instead of using the National Cancer Institute multivariate algorithm as described earlier [47, 48]. Nevertheless, it must be emphasized that the underlying data was gathered with utmost care, using prospective weighed food diaries instead of more error-prone 24 h-dietary recalls. Still, we transparently report the potential presence of measurement error in the underlying data [49].

Despite these limitations [8, 9], this first examination allowed us to start disentangling the complex interplay between nutritional status and nutrient intakes from supplements and foods relevant to healthy individuals adhering to omnivore, ovo-lacto-vegetarian and vegan diets. This study also revealed the need to define diet-specific TNIs, and to use caution when utilizing currently defined TNIs when comparing the nutrient quality of omnivore versus the spectrum of plant-based diets.

## Conclusions

Plant-based diets overall do not fare worse than omnivorous diets when it comes to the dietary intake of eight potentially *under-consumed* nutrients as captured by the TNI (vitamins A, C, D, and E, calcium, magnesium, potassium, and choline). Additionally, *under-consumed* nutrients from foods varied by dietary patterns, suggesting the need for diet-specific TNI scores. Supplementation has a profound impact on nutrient supplies in individuals on a plant-based diet (particularly in vegans, much less in lacto-ovo-vegetarians). The present study thus reiterates the need to quantitatively assess nutrient intakes from supplements when discussing about the diet quality of plant-based dietary patterns. Studies that capture supplements on a mere qualitative basis may underestimate the overall nutrient intake of plant-based individuals.

## Supplementary Information

Supplementary Material 1: Table 1. Adequate intakes (AI) and recommended dietary allowances (RDA) used for the Total Nutrient Index (TNI) assessment in this secondary analysis.

Supplementary Material 2: Table 2. Food Nutrient Index (FNI) and Total Nutrient Index (TNI) by dietary group.

Supplementary Material 3: Figure 1. Deviationplots – Food Nutrient Index (FNI) and Total Nutrient Index (TNI) by dietary group. Values of the FNI and TNI are shown as deviations from the mean/median in increasing order [30]. Each deviation is represented as a vertical spike with base given by the mean or median and with a marker symbol showing the value relative to a vertical scale. Panel a: ANOVA-based deviation plot for the FNI; panel b: ANOVA-based deviation plot for the TNI; panel c: unadjusted deviation plot for median FNI scores; d = unadjusted deviation plot for median TNI scores. Based on n = 108 observations. OM = omnivores; LOV = lacto-ovo-vegetarians; VN = vegans.

Supplementary Material 4: Figure 2. Scatterplots – Associations between the Total Nutrient Index (TNI) and serum levels of Vitamin A, D and E. Scatterplots depict bivariate associations between the TNI and serum levels of Vitamin A, D and E. Based on *n* = 105 observations for vitamin A and vitamin D. *Based on n = 107 observations for vitamin E*. A significant correlation was found for vitamin D (Spearman's rho = 0.45; *p* =<0.001) and vitamin A (Spearman's rho = 0.21; *p* = 0.034), whereas the association with vitamin E was not statistically significant (Spearman's rho = -0.05; *p* = 0.623). OM = omnivores; LOV = lacto-ovo-vegetarians; VN = vegans.

Supplementary Material 5. Code used for calculating the Total Nutrient Index (TNI).

## Data Availability

Data contained in this manuscript will be made available upon reasonable request.

## Abbreviations

DGA: Dietary Guidelines for Americans
FNI: Food Nutrient Index
LOV: Lacto-Ovo-Vegetarians
OM: Omnivores
TNI: Total Nutrient Index
USDA: US Department of Agriculture
VN: Vegans

## References

[1] Kent G, Kehoe L, Flynn A, Walton J. Plant-based diets: a review of the definitions and nutritional role in the adult diet. Proceedings of the Nutrition Society. 2022Mar;81(1):62–74. 35760588 10.1017/S0029665121003839

[2] Storz MA. What makes a plant-based diet? a review of current concepts and proposal for a standardized plant-based dietary intervention checklist. Eur J Clin Nutr. 2022Jun;76(6):789–800. 34675405 10.1038/s41430-021-01023-z PMC9187516

[3] Evolving appetites: an in depth look at European attitudes towards plant based eating. A follow up to the 2021 survey report, ‘What Consumers Want’ European Union’s Horizon 2020 research and innovation programme (No 862957) (2023). https://proveg.org/report/evolving-appetites-an-in-depth-look-at-european-attitudes-towards-plant-based-eating. Accessed on 05 January 2025.

[4] Wang T, Masedunskas A, Willett WC, Fontana L. Vegetarian and vegan diets: benefits and drawbacks. Eur Heart J. 2023Sep 21;44(36):3423–39. 37450568 10.1093/eurheartj/ehad436 PMC10516628

[5] Dean E, Xu J, Jones AYM, Vongsirinavarat M, Lomi C, Kumar P, et al. An unbiased, sustainable, evidence-informed Universal Food Guide: a timely template for national food guides. Nutr J. 2024Oct 18;23(1):126. 39425106 10.1186/s12937-024-01018-z PMC11487974

[6] Dean E, Xu J, Jones AY, Vongsirinavarat M, Lomi C, Kumar P, et al. Correction: An unbiased, sustainable, evidence-informed Universal Food Guide: a timely template for national food guides. Nutr J. 2024Nov 15;23(1):144. 39548484 10.1186/s12937-024-01043-y PMC11566198

[7] Neufingerl N, Eilander A. Nutrient Intake and Status in Adults Consuming Plant-Based Diets Compared to Meat-Eaters: A Systematic Review. Nutrients. 2021Dec 23;14(1):29. 35010904 10.3390/nu14010029 PMC8746448

[8] Storz MA, Müller A, Niederreiter L, Zimmermann-Klemd AM, Suarez-Alvarez M, Kowarschik S, et al. A cross-sectional study of nutritional status in healthy, young, physically-active German omnivores, vegetarians and vegans reveals adequate vitamin B12 status in supplemented vegans. Ann Med. 2023;55(2):2269969. 37851870 10.1080/07853890.2023.2269969 PMC10586079

[9] Correction. Ann Med. 2024 Dec;56(1):2346423. 10.1080/07853890.2024.2346423 PMC11073407 38701001

[10] Storz MA, Huber R, Hannibal L. Impact of vitamin B12 supplement intake cessation on vitamin B12 status in a healthy vegan: A close interval monitoring case study. Nutrition. 2024Sep;1(125): 112498. 10.1016/j.nut.2024.112498 38833779

[11] Bakaloudi DR, Halloran A, Rippin HL, Oikonomidou AC, Dardavesis TI, Williams J, et al. Intake and adequacy of the vegan diet. A systematic review of the evidence. Clinical Nutrition. 2021;40(5):3503–21. 33341313 10.1016/j.clnu.2020.11.035

[12] Craig WJ. Nutrition concerns and health effects of vegetarian diets. Nutr Clin Pract. 2010Dec;25(6):613–20. 21139125 10.1177/0884533610385707

[13] Koeder C, Perez-Cueto FJA. Vegan nutrition: a preliminary guide for health professionals. Crit Rev Food Sci Nutr. 2024Jan 25;64(3):670–707. 35959711 10.1080/10408398.2022.2107997

[14] Soh BXP, Smith NW, von Hurst PR, McNabb WC. Achieving High Protein Quality Is a Challenge in Vegan Diets: A Narrative Review. Nutr Rev. 2024 Dec 11;nuae176. 10.1093/nutrit/nuae176 PMC12166188 39661760

[15] Deutsche Gesellschaft für Ernährung e.V. Vegane Ernährung http://www.dge.de/gesunde-ernaehrung/faq/faqs-vegane-ernaerung/. Accessed on 05 January 2025.

[16] Roeren M, Kordowski A, Sina C, Smollich M. Inadequate Choline Intake in Pregnant Women in Germany. Nutrients. 2022Nov 17;14(22):4862. 36432547 10.3390/nu14224862 PMC9696170

[17] Van Parys A, Brække MS, Karlsson T, Vinknes KJ, Tell GS, Haugsgjerd TR, et al. Assessment of Dietary Choline Intake, Contributing Food Items, and Associations with One-Carbon and Lipid Metabolites in Middle-Aged and Elderly Adults: The Hordaland Health Study. J Nutr. 2022Feb 1;152(2):513–24. 34643705 10.1093/jn/nxab367 PMC8826836

[18] Liu C, Sun X, Peng J, Yu H, Lu J, Feng Y. Association between dietary vitamin A intake from different sources and non-alcoholic fatty liver disease among adults. Sci Rep. 2024Jan 22;14(1):1851. 38253816 10.1038/s41598-024-52077-5 PMC10803811

[19] Cowan AE, Bailey RL, Jun S, Dodd KW, Gahche JJ, Eicher-Miller HA, et al. The Total Nutrient Index is a Useful Measure for Assessing Total Micronutrient Exposures Among US Adults. J Nutr. 2022Mar 3;152(3):863–71. 34928350 10.1093/jn/nxab428 PMC8891182

[20] Cowan AE, Jun S, Tooze JA, Dodd KW, Gahche JJ, Eicher-Miller HA, et al. A narrative review of nutrient based indexes to assess diet quality and the proposed total nutrient index that reflects total dietary exposures. Crit Rev Food Sci Nutr. 2023;63(12):1722–32. 34470512 10.1080/10408398.2021.1967872 PMC8888777

[21] Dawczynski C, Weidauer T, Richert C, Schlattmann P, Dawczynski K, Kiehntopf M. Nutrient Intake and Nutrition Status in Vegetarians and Vegans in Comparison to Omnivores - the Nutritional Evaluation (NuEva) Study. Front Nutr. 2022;9: 819106. 35651513 10.3389/fnut.2022.819106 PMC9149309

[22] Dawczynski C, Weidauer T, Richert C, Schlattmann P, Dawczynski K, Kiehntopf M. Corrigendum: Nutrient intake and nutrition status in vegetarians and vegans in comparison to omnivores-the nutritional evaluation (NuEva) study. Front Nutr. 2022;9: 975159. 35967804 10.3389/fnut.2022.975159 PMC9365051

[23] Storz MA. Does Self-Perceived Diet Quality Align with Nutrient Intake? A Cross-Sectional Study Using the Food Nutrient Index and Diet Quality Score. Nutrients. 2023Jun 12;15(12):2720. 37375624 10.3390/nu15122720 PMC10305402

[24] USDA. Dietary Guidelines for Americans. https://www.dietaryguidelines.gov. Accessed on 05 January 2025.

[25] Deutsche Gesellschaft für Ernährung. Referenzwerte. http://www.dge.de/wissenschaft/referenzwerte. Accessed on 05 January 2025.

[26] Patterson KY, Bhagwat SA, Williams JR, Howe JC, Holden JM. USDA Database for the Choline Content of Common Foods - Release Two. https://agdatacommons.nal.usda.gov/articles/dataset/USDA_Database_for_the_Choline_Content_of_Common_Foods_Release_2_2008_/24660123. Accessed on 05 January 2025.

[27] USDA. Nutrient Lists from Standard Reference Legacy (2018). https://www.nal.usda.gov/human-nutrition-and-food-safety/nutrient-lists-standard-reference-legacy-2018. Accessed on 05 January 2025.

[28] Richard C, Lewis ED, Zhao YY, Asomaning J, Jacobs RL, Field CJ, et al. Measurement of the total choline content in 48 commercial dairy products or dairy alternatives. J Food Compos Anal. 2016Feb;1(45):1–8.

[29] Cox N. STRIPPLOT: Stata module for strip plots (one-way dot plots)," Statistical Software Components S433401, Boston College Department of Economics, 2003, revised 05 May 2024.

[30] Cox N. DEVNPLOT: Stata module for deviation plots. Statistical Software Components S457233, Boston College Department of Economics, 2011, revised 19 Mar 2014.

[31] Nolden AA, Forde CG. The Nutritional Quality of Plant-Based Foods. Sustainability. 2023Jan;15(4):3324.

[32] Tallman DA, Khor BH, Karupaiah T, Khosla P, Chan M, Kopple JD. Nutritional Adequacy of Essential Nutrients in Low Protein Animal-Based and Plant-Based Diets in the United States for Chronic Kidney Disease Patients. J Ren Nutr. 2023Mar 1;33(2):249–60. 36460269 10.1053/j.jrn.2022.10.007

[33] Dressler J, Storz MA, Müller C, Kandil FI, Kessler CS, Michalsen A, et al. Does a Plant-Based Diet Stand Out for Its Favorable Composition for Heart Health? Dietary Intake Data from a Randomized Controlled Trial. Nutrients. 2022Nov 1;14(21):4597. 36364858 10.3390/nu14214597 PMC9656677

[34] Bundesministerium für Ernährung und Landwirtschaft. Deutschland, wie es isst - der BMEL-Ernährungsreport 2023. https://www.bmel.de/DE/themen/ernaehrung/ernaehrungsreport2023.html. Accessed on 05 January 2025.

[35] Satija A, Bhupathiraju SN, Spiegelman D, Chiuve SE, Manson JE, Willett W, et al. Healthful and Unhealthful Plant-Based Diets and the Risk of Coronary Heart Disease in U.S. Adults. Journal of the American College of Cardiology. 2017;70(4):411–22. 28728684 10.1016/j.jacc.2017.05.047 PMC5555375

[36] Herter J, Stübing F, Lüth V, Zimmermann J, Lederer AK, Hannibal L, et al. Bowel health, defecation patterns and nutrient intake following adoption of a vegan diet: a randomized-controlled trial. Ann Med. 2024Dec;56(1):2305693. 38327148 10.1080/07853890.2024.2305693 PMC10854443

[37] U.S. Agency for International Development. USAID Food Fortification: Delivering Essential Nutrients for a Healthy Diet. https://www.usaid.gov/global-health/resources/fact-sheets/food-fortification. Accessed on 05 January 2025.

[38] Jaiswal A, Dewani D, Reddy LS, Patel A. Choline Supplementation in Pregnancy: Current Evidence and Implications. Cureus. 15(11):e48538. 10.7759/cureus.48538 PMC10709661 38074049

[39] Office of Dietary Supplements. Choline. Available from: https://ods.od.nih.gov/factsheets/Choline-HealthProfessional. Accessed on 05 January 2025.

[40] Derbyshire E. Could we be overlooking a potential choline crisis in the United Kingdom? BMJ Nutrition, Prevention & Health. 2019 Aug 29;bmjnph. 10.1136/bmjnph-2019-000037 PMC7664488 33235962

[41] Zhu W, Wang Z, Tang WHW, Hazen SL. Gut Microbe-Generated Trimethylamine N-Oxide From Dietary Choline Is Prothrombotic in Subjects. Circulation. 2017Apr 25;135(17):1671–3. 28438808 10.1161/CIRCULATIONAHA.116.025338 PMC5460631

[42] Tang WHW, Wang Z, Levison BS, Koeth RA, Britt EB, Fu X, et al. Intestinal microbial metabolism of phosphatidylcholine and cardiovascular risk. N Engl J Med. 2013Apr 25;368(17):1575–84. 23614584 10.1056/NEJMoa1109400 PMC3701945

[43] Rosner B, Gore R. Measurement Error Correction in Nutritional Epidemiology based on Individual Foods, with Application to the Relation of Diet to Breast Cancer. Am J Epidemiol. 2001Nov 1;154(9):827–35. 11682365 10.1093/aje/154.9.827

[44] Bailey RL, Dodd KW, Gahche JJ, Dwyer JT, Cowan AE, Jun S, et al. Best Practices for Dietary Supplement Assessment and Estimation of Total Usual Nutrient Intakes in Population-Level Research and Monitoring. J Nutr. 2019Feb 1;149(2):181–97. 30753685 10.1093/jn/nxy264 PMC6374152

[45] Tooze JA, Kipnis V, Buckman DW, Carroll RJ, Freedman LS, Guenther PM, et al. A mixed-effects model approach for estimating the distribution of usual intake of nutrients: The NCI method. Stat Med. 2010;29(27):2857–68. 20862656 10.1002/sim.4063 PMC3865776

[46] Dekkers AL, Verkaik-Kloosterman J, van Rossum CT, Ocké MC. SPADE, a New Statistical Program to Estimate Habitual Dietary Intake from Multiple Food Sources and Dietary Supplements. J Nutr. 2014Dec 1;144(12):2083–91. 25320187 10.3945/jn.114.191288

[47] Zhang S, Krebs-Smith SM, Midthune D, Perez A, Buckman DW, Kipnis V, et al. Fitting a bivariate measurement error model for episodically consumed dietary components. Int J Biostat. 2011;7(1):1. 22848190 10.2202/1557-4679.1267 PMC3406506

[48] Zhang S, Carroll RJ, Midthune D, Guenther PM, Krebs-Smith SM, Kipnis V, et al. A new multivariate measurement error model with zero-inflated dietary data, and its application to dietary assessment. The Annals of Applied Statistics. 2011Jun;5(2B):1456–87. 21804910 10.1214/10-AOAS446 PMC3145332

[49] Brakenhoff TB, Mitroiu M, Keogh RH, Moons KGM, Groenwold RHH, van Smeden M. Measurement error is often neglected in medical literature: a systematic review. J Clin Epidemiol. 2018Jun;1(98):89–97. 10.1016/j.jclinepi.2018.02.023 29522827

