# Supplementary material for: Analyzing dietary exposure to critical nutrients on a plant-based diet using the food- and total nutrient index
Source: Nutr J. 2025 Mar 12;24:39. doi: 10.1186/s12937-025-01105-9 (PMC11899309; doi:10.1186/s12937-025-01105-9)
Supplement: Supplementary file 1 — Supplementary Material 1: Table 1. Adequate intakes (AI) and recommended dietary allowances (RDA) used for the Total Nutrient Index (TNI) assessment in this secondary analysis. [file 12937_2025_1105_MOESM1_ESM.docx]

# Supplementary Tables

## Supplementary Table 1

**Supplementary Table 1. Adequate intakes (AI) and recommended dietary allowances (RDA) used for the Total Nutrient Index (TNI) assessment in this secondary analysis**

| TNI-relevant nutrient | Source of Goal | Males | | | Females | |
| --- | --- | --- | --- | --- | --- | --- |
|  | | |  |  |  |  |
|  |  | 19–30 years | | 31–50 years | 19–30 years | 31–50 years |
|  | | |  |  |  |  |
| Calcium | RDA | 1000 mg/d | | 1000 mg/d | 1000 mg/d | 1000 mg/d |
| Magnesium | RDA | 400 mg/d | | 420 mg/d | 310 mg/d | 320 mg/d |
| Potassium | AI | 3400 mg/d | | 3400 mg/d | 2600 mg/d | 2600 mg/d |
| Choline | AI | 550 mg/d | | 550 mg/d | 425 mg/d | 425 mg/d |
| Vitamin A | RDA | 900 mcg RAE/d | | 900 mcg RAE/d | 700 mcg RAE/d | 700 mcg RAE/d |
| Vitamin C | RDA | 90 mg/d | | 90 mg/d | 75 mg/d | 75 mg/d |
| Vitamin D | RDA | 600 IU/d | | 600 IU/d | 600 IU/d | 600 IU/d |
| Vitamin E | RDA | 15 mg/d | | 15 mg/d | 15 mg/d | 15 mg/d |

Supplementary Table 1 legend: AI = Adequate Intakes. RAE = Retinol Activity Equivalents. RDA = Recommended Dietary Allowance. Supplementary Table 1 lists the age and sex-specific nutrient intake recommendations (daily nutritional goals) used in this study. Recommendations were obtained from the Dietary Guidelines for Americans [24].
