# Supplementary material for: Analyzing dietary exposure to critical nutrients on a plant-based diet using the food- and total nutrient index
Source: Nutr J. 2025 Mar 12;24:39. doi: 10.1186/s12937-025-01105-9 (PMC11899309; doi:10.1186/s12937-025-01105-9)
Supplement: Supplementary file 2 — Supplementary Material 2: Table 2. Food Nutrient Index (FNI) and Total Nutrient Index (TNI) by dietary group. [file 12937_2025_1105_MOESM2_ESM.docx]

## Supplementary Table 2

**Supplementary Table 2. Food Nutrient Index (FNI) and Total Nutrient Index (TNI) by dietary group:**

|  | **Omnivores (n=40)** | **Lacto-Ovo-Vegetarians (n=33)** | **Vegans (n=35)** | **p-value** |
| --- | --- | --- | --- | --- |
|  |  |  |  |  |
| FNI (0-100) | 63.04 ± 16.57 | 62.05 ± 13.89 | 62.87 ± 14.24 | 0.954 ^a^ |
| TNI (0-100) | 73.70 ± 19.68 | 68.50 ± 17.10 | 72.77 ± 17.88 | 0.792 ^a^ |
|  |  |  |  |  |
|  | **Omnivores (n=20):**  **19-30 years** | **Lacto-Ovo-Vegetarians (n=25):**  **19-30 years** | **Vegans (n=25):**  **19-30 years** |  |
| FNI (0-100) | 58.49 ± 14.23 | 60.46 ± 13.88 | 63.41 ± 12.14 | 0.402 ^b^ |
| TNI (0-100) | 69.89 ± 17.87 | 65.47 ± 15.78 | 74.43 ± 15.78 | 0.212 ^b^ |
|  |  |  |  |  |
|  | **Omnivores (n=20):**  **31-50 years** | **Lacto-Ovo-Vegetarians (n=8):**  **31-50 years** | **Vegans (n=10):**  **31-50 years** |  |
| FNI (0-100) | 67.58 ± 17.82 | 67.03 ± 13.58 | 61.52 ± 19.24 | 0.568 ^b^ |
| TNI (0-100) | 82.40 (37.53) | 77.96 ± 18.66 | 68.62 ± 22.72 | 0.465 ^b^ |

Supplementary Table 2 legend: Normally distributed data is shown with its mean ± standard deviation; not normally distributed data is shown with its median and IQR in parenthesis. ^a^ = based on analysis of variance^b^ = based on Kruskal-Wallis H test (selected in light of subpopulation sizes n < 30)
