# Supplementary figures and images for: Analyzing dietary exposure to critical nutrients on a plant-based diet using the food- and total nutrient index
Source: Nutr J. 2025 Mar 12;24:39. doi: 10.1186/s12937-025-01105-9 (PMC11899309; doi:10.1186/s12937-025-01105-9)

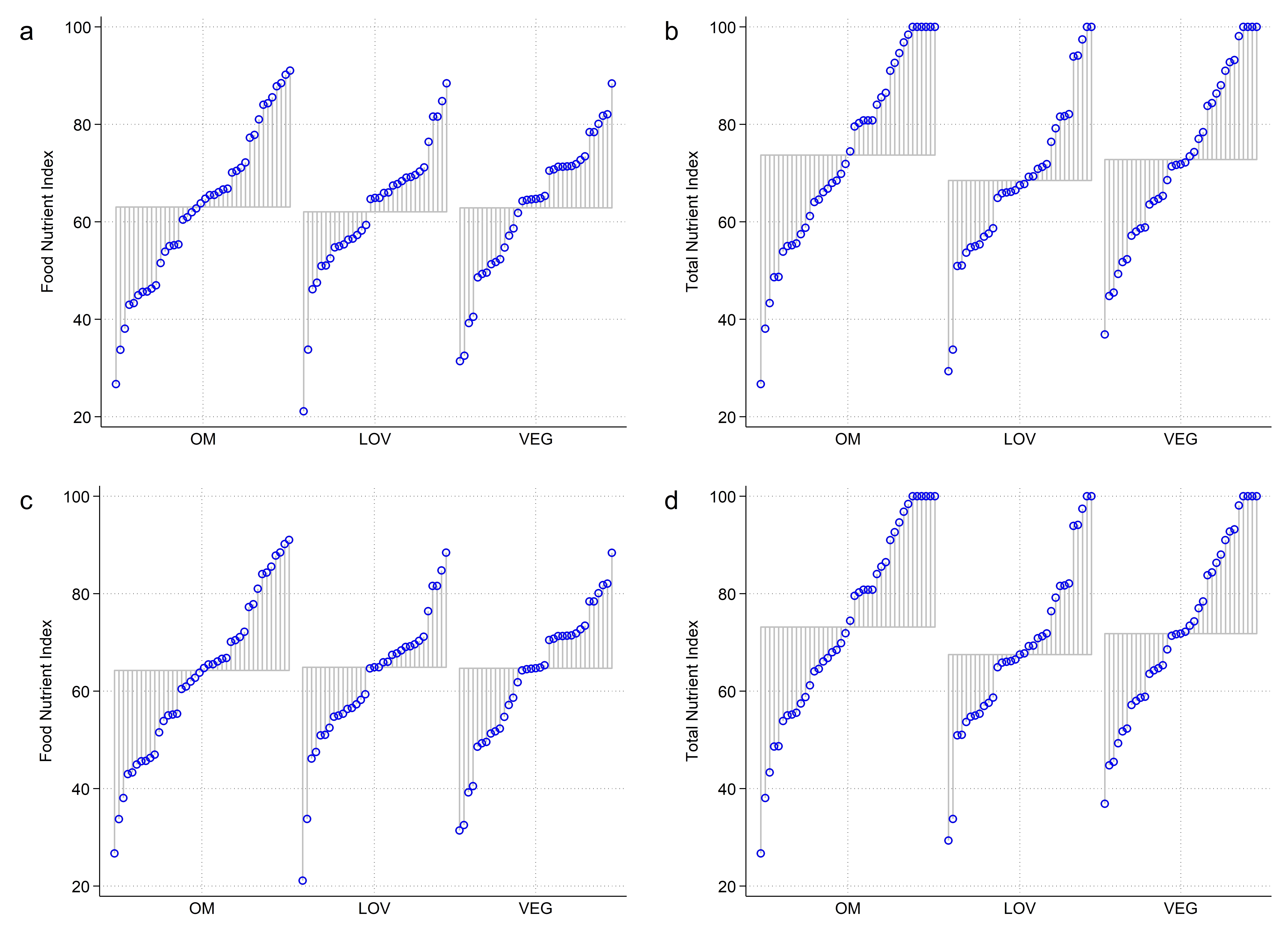

Supplement: Supplementary file 3 — Supplementary Material 3: Figure 1. Deviationplots – Food Nutrient Index (FNI) and Total Nutrient Index (TNI) by dietary group. Values of the FNI and TNI are shown as deviations from the mean/median in increasing order [30]. Each deviation is represented as a vertical spike with base given by the mean or median and with a marker symbol showing the value relative to a vertical scale. Panel a: ANOVA-based deviation plot for the FNI; panel b: ANOVA-based deviation plot for the TNI; panel c: unadjusted deviation plot for median FNI scores; d = unadjusted deviation plot for median TNI scores. Based on n = 108 observations. OM = omnivores; LOV = lacto-ovo-vegetarians; VN = vegans. [file 12937_2025_1105_MOESM3_ESM.jpg]

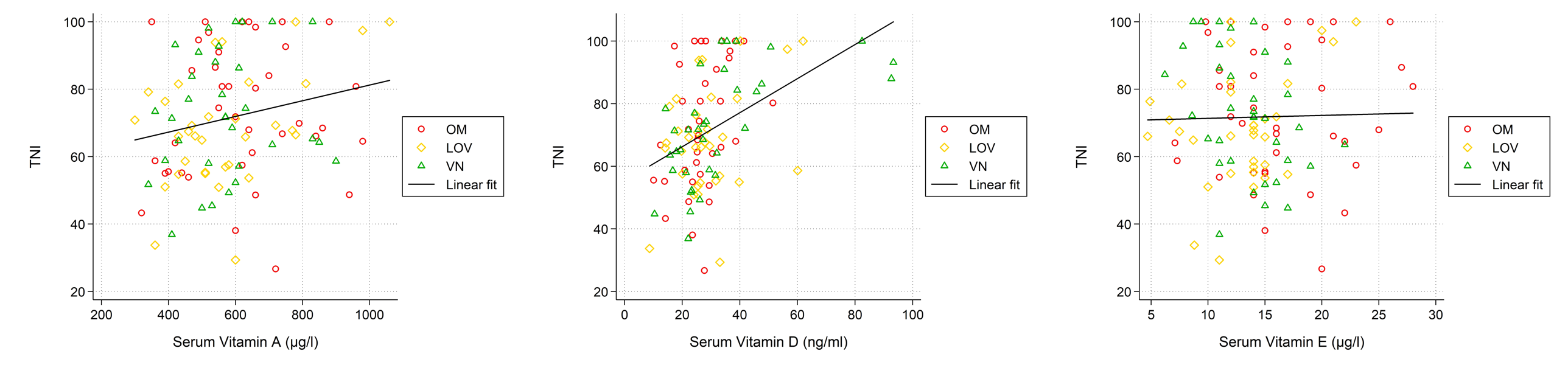

Supplement: Supplementary file 4 — Supplementary Material 4: Figure 2. Scatterplots – Associations between the Total Nutrient Index (TNI) and serum levels of Vitamin A, D and E. Scatterplots depict bivariate associations between the TNI and serum levels of Vitamin A, D and E. Based on n = 105 observations for vitamin A and vitamin D. Based on n = 107 observations for vitamin E. A significant correlation was found for vitamin D (Spearman's rho = 0.45; p =<0.001) and vitamin A (Spearman's rho = 0.21; p = 0.034), whereas the association with vitamin E was not statistically significant (Spearman's rho = -0.05; p = 0.623). OM = omnivores; LOV = lacto-ovo-vegetarians; VN = vegans. [file 12937_2025_1105_MOESM4_ESM.jpg]
